# The Novel Pro-Osteogenic Activity of NUCB2^1–83^

**DOI:** 10.1371/journal.pone.0061619

**Published:** 2013-04-15

**Authors:** Ruishu Li, Qiong Wu, Yue Zhao, Wenbo Jin, Xinfang Yuan, Xiaopeng Wu, Yanchun Tang, Jing Zhang, Xiangyang Tan, Feng Bi, Jian-Ning Liu

**Affiliations:** 1 Institute of Molecular Medicine, Nanjing University, Nanjing, China; 2 Landing Laboratories, Suzhou, China; University of California Los Angeles, United States of America

## Abstract

NUCB2^1–83^ has been recently reported as an anorexigenic and anti-hyperglycemic peptide. Here we report that NUCB2^1–83^ promotes osteogenesis. It was found after two months of once-a-day intravenous injection of NUCB2^1–83^, bone mineral density of femora and lumbar vertebrae were increased in ovariectomized rats. NUCB2^1–83^ also increased the alkaline phosphatase activity and promoted mineralization in mouse MC3T3-E1 preosteoblastic cell line. When either both Arg^60^ and Arg^63^ or Ser^72^ were mutated to Ala, the pro-osteogenic activity was completely lost, indicating that these residues are structurally important for its biological function. Furthermore, it encumbered osteoclastic differentiation of RAW 264.7 macrophage. It also excluded any possibility of the effect caused by contaminants or experimental faults, and demonstrated that the pro-osteogenic activity observed was a specific effect of NUCB2^1–83^ itself. These findings warranted that further studies on NUCB2^1–83^ would be valuable for the treatment of bone metabolic diseases especially for osteoporosis.

## Introduction

Osteoporosis is a skeletal disease characterized by loss of bone mass and strength that leads to fractures [1]. It is a public health threat due to the potentially disastrous results [2] and high cumulative rate of fractures. It is estimated that more than 100 million people worldwide are at risk for the disorder [3] and fracture rates seem to be rising ceaselessly.

Osteoporosis is mainly caused by an imbalance between osteoblast-mediated bone formation and osteoclast-mediated bone resorption [4], [5]. A number of medicines have been developed to treat osteoporosis, mainly including (1) bone resorption inhibitors, which prevent excessive bone loss by reducing the osteoclast formation and activity; (2) bone formation accelerators, which increase bone mineral density and bone mass by stimulating the osteoblast activity; (3) bone mineralization drugs, which stimulate new bone mineralization.

NUCB2^1–83^ is also called nesfatin-1. It has recently been identified as a satiety molecule associated with melanocortin signaling system detectable in central neurons [6] as well as an anti-hyperglycemic peptide when it is given intravenously [7]. NUCB2^1–83^ was also reported to have a role in the response to stress and mediation of anxiety- and/or fear-related behaviors in rats [8], [9]. The expression of NUCB2^1–83^ was induced by troglitazone, an activator of peroxisome proliferator-activated receptor-γ (PPAR-γ) [6]. The activation of PPAR-γ was recognized to cause loss of bone [10]. Therefore, we have curiously examined the effect of NUCB^1–83^ on bone metabolism.

Since ovariectomized (OVX) rat is a classic animal model for postmenopausal osteoporosis, we have intravenously (i.v.) injected NUCB2^1–83^ once a day to OVX rats continuously for two months to observe the changes in bone mineral density (BMD). In addition, we have also evaluated both the promoting effect of NUCB2^1–83^ on osteoblastogenesis in the mouse MC3T3-E1 preosteoblastic cell line and its inhibitory effect on osteoclastogenesis in murine RAW 264.7 macrophages, as well as its presence in osteoblasts and osteoclasts.

## Materials and Methods

### Materials and Reagents

Recombinant human bone morphogenetic protein-2 (rhBMP-2) was prepared by our laboratory [11], as well as recombinant NUCB2^1–83^ and its mutants. No endotoxin was detected in the preparation of recombinant NUCB2^1–83^ using a Limulus Amoebocyte Lysate test kit. The BCA protein assay kit was purchased from Pierce (Rockford, IL, USA). The ALP activity kit and the commercial calcium kit were obtained from Nanjing Jiancheng Biological Institute (Nanjing, China). Leukocyte acid phosphatase kit was purchased from Sigma-Aldrich (St. Louis, MO). MC3T3-E1 cells and RAW 264.7 cells were obtained from CCTCC (Wuhan, China).

### Animal Experiments

Twenty 3-month-old virgin female Sprague-Dawley rats were obtained from the Experimental Animal Centre of Qinglongshan (Nanjing, China). All rats were housed at 25°C with a 12-h light/dark cycle with lab chow and water available *ad libitum*. All procedures in animal experiments were conducted with the approval from the Animal Study Committee of Institute of Molecular Medicine, Nanjing University *(Study Number: IMM022-10)*. Rats were ovariectomized or sham-operated. Some animals were euthanized at 16 weeks after surgery, and femora and lumbar vertebrae were dissected free of soft tissues and stored in 75% ethanol for the BMD assay. The ovariectomized rats were then divided into two groups and intravenously injected with saline or NUCB2^1–83^ (50 nmol) once a day. After 60 days, their femora and lumbar vertebrae were collected for the BMD assay as described below.

### Bone Densitometry

BMD were measured for femora of both sides and lumbar vertebrae using a dual energy X-ray absorptiometry (QDR 2000 Plus; Histologic, Bedford, MA, USA).

### MC3T3-E1 Cell Cultures

For ALP Assay, MC3T3-E1 cells were seeded at a density of 2×10^5^ cells/30-mm well in α-modified minimal essential medium (α-MEM, GIBCO, Grand Island, NY, USA) containing 10% fetal bovine serum (FBS, GIBCO) supplemented with 100 U/mL penicillin (North China Pharmaceutical, Shijiazhuang, China) and 100 U/mL streptomycin (Lu-Kang, Jining, China) in 5% CO_2_ at 37°C. Twenty-four hours after plating, the adherent cells were treated with 1 µg/mL of rhBMP-2. Simultaneously, test samples were added to the cells respectively. The conditioned medium was changed on the third day after the addition of the test samples.

On the sixth day, the cells were harvested by trypsinization and suspended in 1 mL of phosphate buffered saline (PBS). The cell suspensions were sonicated in ice bath for 1 min for protein determination and the ALP assay.

For mineralization assay, MC3T3-E1 cells were cultured in 10 cm dishes at an initial density of 1×10^5^ cells/cm^2^ in α-MEM containing 10% FBS and 100 U/mL penicillin and 100 U/mL streptomycin in 5% CO_2_ at 37°C. To support differentiation, 10 mM β-glycerophosphate (Sinopham Chemical Reagent, Shanghai, China) and 50 µg/mL ascorbic acid (Sinopham Chemical Reagent) were added to the cultures. 50 nM NUCB2^1–83^ was added into the conditioned medium in experimental group and the medium was then changed twice a week. The control and experimental groups were cultured for 15 days.

### Alkaline Phosphatase (ALP) Assay

After sonication, the alkaline phosphatase activity of the lysates was measured spectrophotometrically using the ALP activity kit. Protein concentration of the lysates was determined by BCA protein assay.

In this experiment, the ALP activity was evaluated by the following formula:
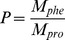



Where P is the ALP activity, M_phe_ is the mass of phenol which can be measured directly and M_pro_ is total protein mass of the sample after digestion and sonication.

### Mineralization Assay

MC3T3-E1 cells were cultured for 15 days and the mineralized matrix was stained by Alizarin-red staining method. After 15 days, cells were washed with PBS and fixed in ice cold 95% ethanol for at least 1 hour. Ethanol was removed, and cells were rinsed with deionized water and stained with 1% (w/v) Alizarin red (Sinopham Chemical Reagent, Shanghai) for 30 minutes at 37°C. Stained cells were further rinsed three times with deionized water and washed for 15 minutes in PBS with rotation to reduce nonspecific Alizarin red stain. Stained cultures were photographed. An appropriate amount of lysate was collected for calcium measurement using the commercial calcium kit.

### RAW 264.7 Cell Culture and Osteoclast Differentiation

RAW 264.7 cells were cultured in DMEM (GIBCO, Grand Island, NY, USA) containing 10% FBS, 2 mM glutamine, 100 U/mL penicillin and 100 U/mL streptomycin in 5% CO_2_ at 37°C. For the osteoclast differentiation, cells were seeded on 24-well plates and grown to about 90% confluence in α-MEM containing 50 ng/mL NF-kappaB ligand (RANKL) (Sigma-Aldrich, St. Louis, MO). 50 nM NUCB2^1–83^ was added into the culture medium in experimental group and the medium were changed every 2 days. The control and experimental groups were cultured for 5 days.

### TRAP (Tartrate-Resistant Acid Phosphatase) Assay

After 5 days of differentiation, RAW 264.7 cells were fixed with 4% formalin for 10 min and stained using leukocyte acid phosphatase kit (Sigma-Aldrich, St. Louis, MO), according to the manufacturer’s instructions. TRAP-positive multinucleated cells were visualized under light microscopy.

For measuring the TRAP activity, cells were fixed with 4% formalin for 10 min and 95% ethanol for 1 min. Subsequently, the dried cells were incubated in 10 mM citrate buffer (pH 4.6) containing 10 mM sodium tratrate (Sigma-Aldrich St. Louis, MO) and 5 mM p-nitrophenylphosphate (New England BioLabs, Beverly, MA, USA ). After incubation for 1 h, the reaction mixtures were transferred into new well plates containing an equal volume of 0.1 N NaOH. Absorbance was measured atλ = 405 nm and the TRAP activity was expressed as percent of that of untreated control.

### RT-PCR and Quantification of NUCB2^1–83^ mRNA in Osteoblasts and Osteoclasts

Total RNA was extracted from MC3T3-E1 cells and differentiated RAW 264.7 cells using the Trizol RNA isolation reagent (Takara, Japan). RNA purity was checked by optical density (OD) absorption ratio (OD260_nm_/OD280_nm_) using a UV-1700 spectrophotometer (SHIMADZU, Japan). One microgram total RNA was added to the RT reaction using AMV reverse transcriptase and an oligo (deoxythymidine) 12–18 primer (Takara, Japan) according to the manufacturer’s protocol. The sequences for the sense and antisense primers (respectively) were:

mouse *NUCB2^1–83^* sense, 5′-ACAAAATGCAGAGGACGATA-3′ and

antisense, 5′-CTCGGTGAATAACTGTTGCT-3′;

mouse *beta-actin* sense, 5′-AGGCCAACCGTGAAAAGATG-3′ and

antisense, 5′-ACCAGAGGCATACAGGGACAA-3′.

SYBR Green real-time PCR was performed on a CFX96™ Real-Time System (Bio-rad, USA) using the primers listed above. The preparations lacking cDNA templates were set as negative controls for the reactions. Protocol conditions consisted of denaturation of 95°C for 300 sec, followed by 45 cycles of 95°C for 15 sec, 60°C for 60 sec, followed by melting-curve analysis. For analysis, expression of *NUCB2^1–83^* was normalized against the expression of the housekeeping gene beta-actin.

### Statistical Analysis

All the results were expressed as the mean±SEM. Statistical significance was evaluated by Student’s *t*-test in BMD assay, calcium measurement and TRAP activity measurement. Statistical significance of ALP assay was determined by one-way analysis of variance (ANOVA) followed by Dunnett's test. A value of P<0.05 was considered to be significant. Statistical analyses were done using GraphPad Prism 5.

## Results

### A Significant Reduction in BMD in OVX Rats

**Figure 1:**
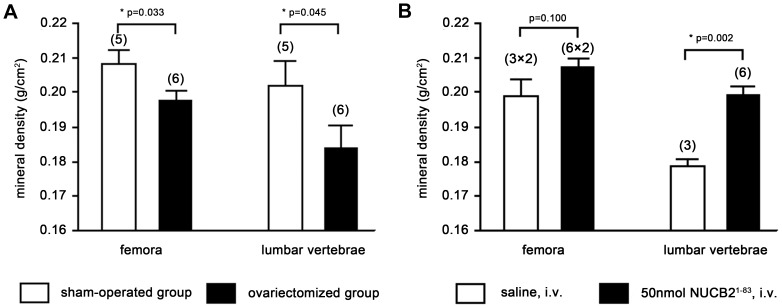
BMD of femora and lumbar vertebrae in the OVX model and experiment groups. (A) BMD in OVX and sham-operated groups. Three-month-old virgin female Sprague-Dawley rats were ovariectomized or sham-operated. They were euthanized at 16 weeks after surgery, and their femora and lumbar vertebrae were dissected free of soft tissues and stored in 75% ethanol for the BMD assay. (B) BMD in different experimental groups after 60-day continuous administration. The ovariectomized rats were divided into two groups and intravenously injected with saline or NUCB2^1–83^ (50 nmol) once a day. After 60 days, their femora and lumbar vertebrae were collected for the BMD assay. Data represented the mean±SEM(*P<0.05). Number of bone samples used is showed within the parentheses.

BMD of femora and lumbar vertebrae in OVX rats were significantly lower than those in the sham-operated controls at 16 weeks after surgery (Fig. 1A). It suggested that the osteoporosis model was established for the study of the effect of NUCB2^1–83^ on bone metabolism.

### The Intravenous Injection of NUCB2^1–83^ Increased BMD in OVX Rats

It was encouraging to notice that BMD of femora and lumbar vertebrae in the NUCB2^1–83^-injected group were higher than those in the control group (Fig. 1B). After 60-day once-per-day administration, BMD of lumbar vertebrae increased about 10% in the NUCB2^1–83^-injected group, which showed significant difference from that in the control group. BMD of femora increased about 5% in the experimental group, while the difference of femoral BMD between the two groups was statistically insignificant. No significant difference was observed in body weight as well as any noticeable side effects during the 60-day administration of NUCB^1–83^.

### NUCB2^1–83^ Significantly Increased the ALP Activity of MC3T3-E1 at the Presence of rhBMP-2

**Figure 2:**
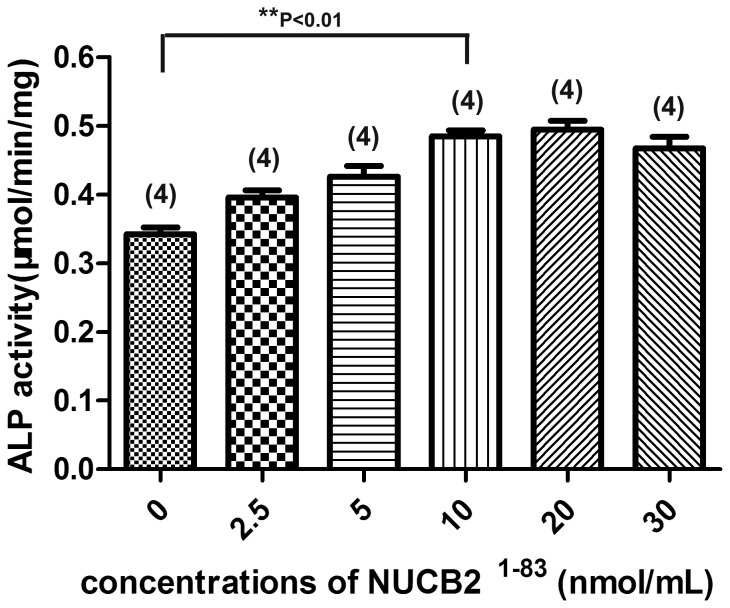
Dose-responsive effects of NUCB2^1–83^ on the ALP activity of MC3T3-E1. MC3T3-E1 cells were treated with test samples for six days in the presence of rhBMP-2 (1 µg/mL). Data represented the mean±SEM. Number of samples used is showed within the parentheses. Statistical analysis according to one-way ANOVA was followed by Dunnett's test (**P<0.01).

In order to investigate the dose-responsive effect of NUCB2^1–83^ on ALP activity of MC3T3-E1 cells, different concentrations of the peptide were added to the conditioned medium containing rhBMP-2 (1 µg/mL). It was demonstrated that NUCB2^1–83^ significantly increased the ALP activity at the concentration of 10 nmol/mL (P<0.01) (Fig. 2, Bar 4). When the concentration of NUCB2^1–83^ was less than 10 nmol/mL, it is linearly correlated to the ALP activity. However, the ALP activity seemed to be independent of NUCB2^1–83^ concentrations above 10 nmol/mL (Fig. 2).

**Figure 3:**
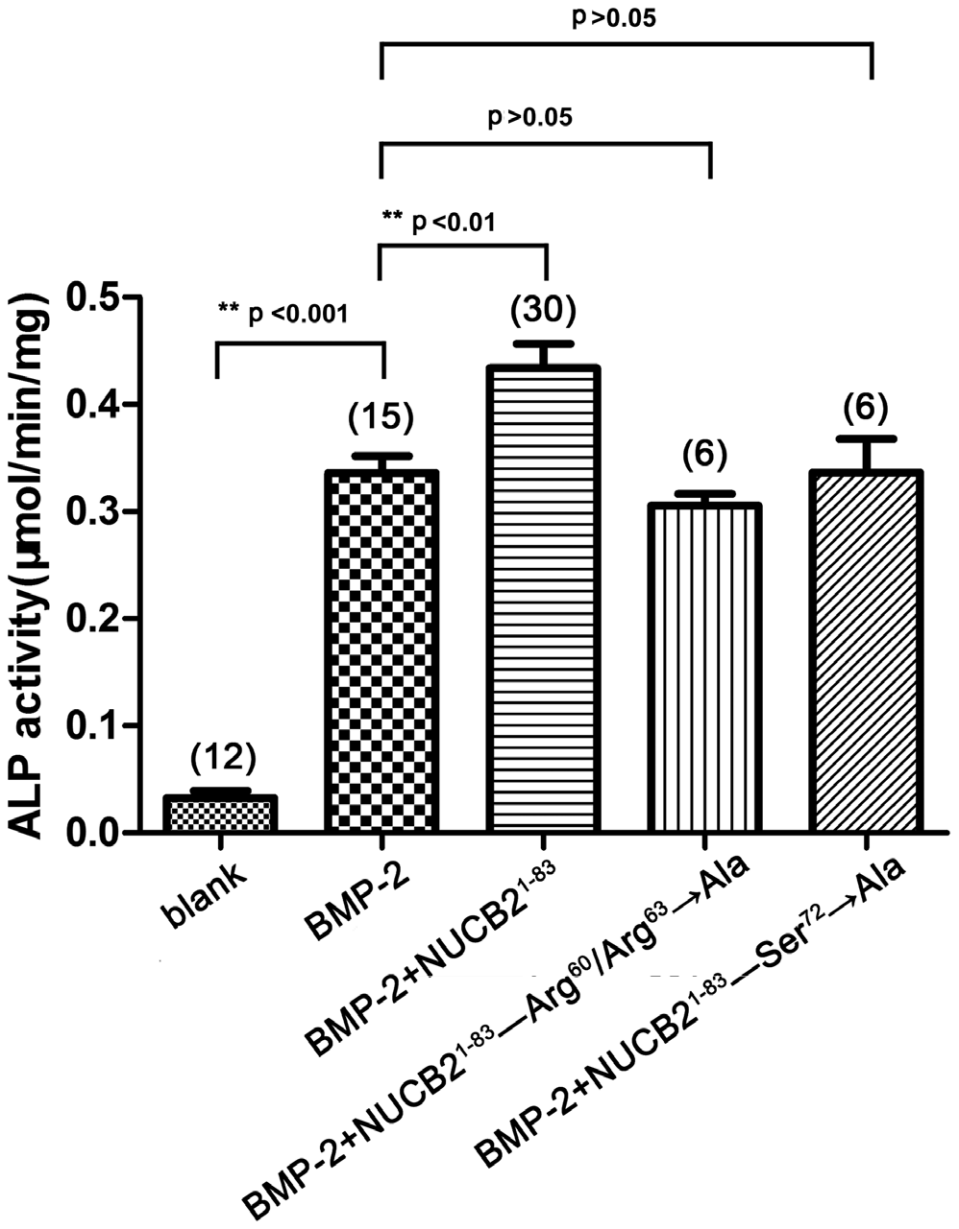
The effect of NUCB2^1–83^ and its mutants on the ALP activity of MC3T3-E1. MC3T3-E1 cells were treated with test samples for six days in the presence of rhBMP-2 (1 µg/mL). The concentrations of NUCB2^1–83^ and its mutants were 10 nmol/mL. Data represented the mean±SEM. Number of samples used is showed within the parentheses. Statistical analysis according to one-way ANOVA was followed by Dunnett's test for BMP-2 vs. test samples (**P<0.01, ***P<0.001).

Next, 10 nmol/mL of NUCB2^1–83^ as well as its mutants were tested to investigate their effects on ALP activity in MC3T3-E1 cells in the presence of 1 µg/mL of rhBMP-2. It was found that 1 µg/mL rhBMP-2 significantly improved the ALP activity of MC3T3-E1 cells (Fig. 3, Bar 2). In the presence of rhBMP-2, NUCB2^1–83^ further increased the ALP activity (Fig. 3, Bar 3), while NUCB2^1–83^(Arg^60^/Arg^63^→Ala) and NUCB2^1–83^(Ser^72^→Ala) lost this promoting effect (Fig. 3, Bar 4, 5). It suggested that Arg^60^/Arg^63^ and Ser^72^ were structurally important for the pro-osteogenic activity of NUCB2^1–83^. These two mutants also served as negative controls, indicating that the promoting activity was a specific effect of NUCB2^1–83^ itself.

### NUCB2^1–83^ Promoted Mineralization of MC3T3-E1

**Figure 4:**
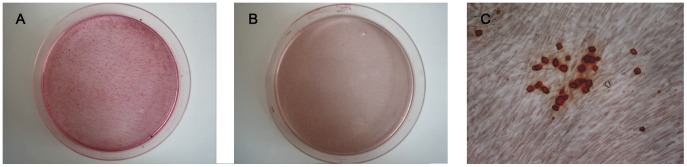
The effect of NUCB2^1–83^ on mineralization measured by Alizarin Red staining. MC3T3-E1 cells were cultured for 15 days with or without NUCB2^1–83^ and the mineralized matrices were stained by Alizarin-red staining method (A) NUCB2^1–83^ (B) control group just with conditional medium (C) The mineralized nodules were observed by microscope *(magnification 100×).*

**Figure 5:**
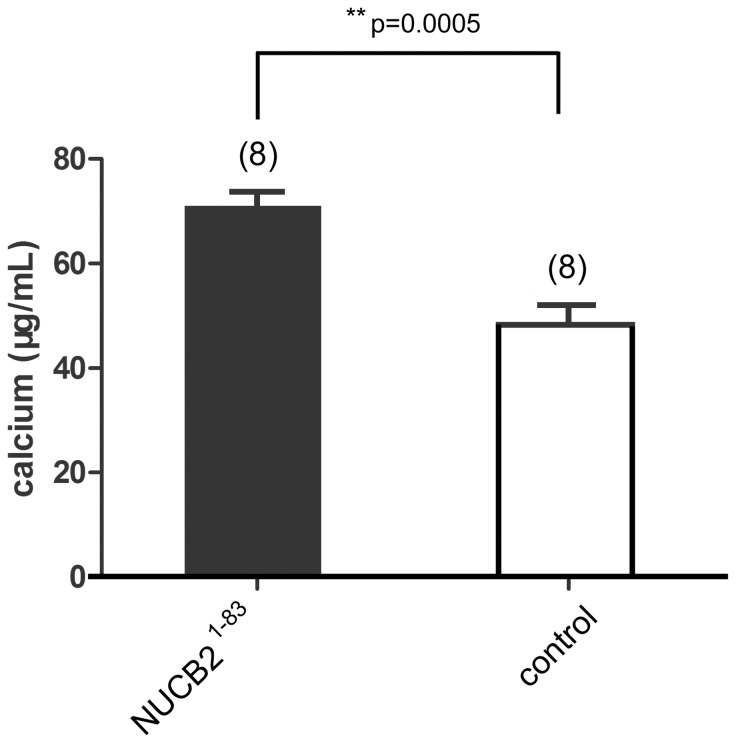
The effect of NUCB2^1–83^ on Mineralization quantified by measuring calcium levels. MC3T3-E1 cells were cultured for 15 days with or without NUCB2^1–83^ and lysates were collected for calcium measurement using the commercial calcium kit. Data represented the mean±SEM(**P<0.01). Number of samples used is showed within the parentheses.

The mineralized nodules and calcium level were obviously increased by the addition of NUCB2^1–83^ (Fig. 4, 5), suggesting that NUCB2^1–83^ promoted mineralization of MC3T3-E1.

### NUCB2^1–83^ Decreased Osteoclastogenesis of RAW 264.7 Stimulated by RANKL

**Figure 6:**
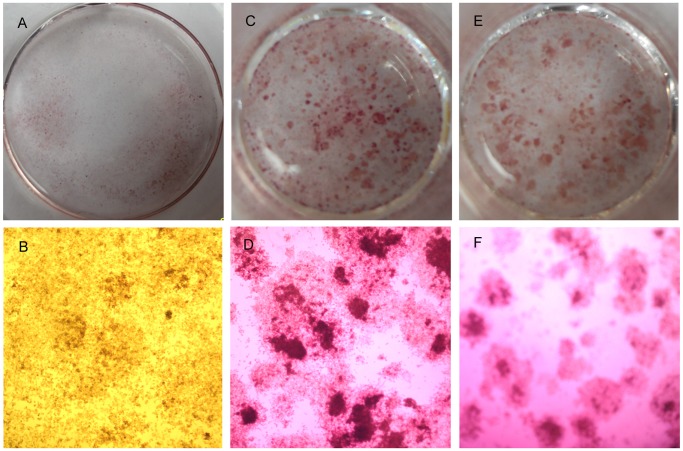
The effect of NUCB2^1–83^ on osteoclastogenesis of RAW 264.7 measured by TRAP staining. RAW 264.7 cells were cultured in the presence of RANKL (50 ng/mL) for 5 days with or without NUCB2^1–83^ and stained for TRAP using leukocyte acid phosphatase kit. TRAP-positive multinucleated cells were visualized under light microscopy. (A,B) control group just with α-MEM (C,D) RANKL (E,F) RANKL and NUCB2^1–83^. B, D, F were taken at 1000× magnification.

**Figure 7:**
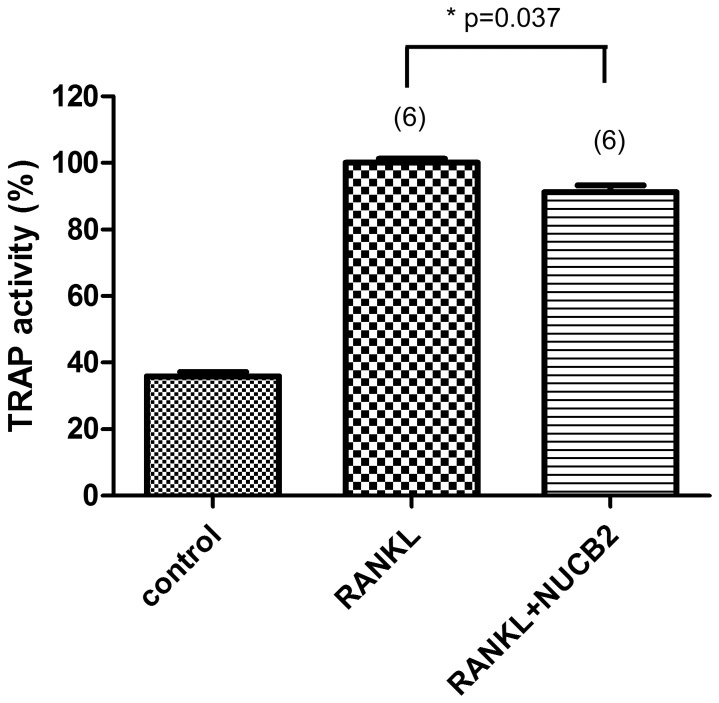
The effect of NUCB2^1–83^ on osteoclast differentiation quantified by measuring TRAP activity. RAW 264.7 cells were cultured in the presence of RANKL (50 ng/mL) for 5 days with or without NUCB2^1–83.^ The cells were fixed and incubated in 10 mM citrate buffer (pH 4.6) containing 10 mM sodium tratrate and 5 mM p-nitrophenylphosphate for 1 h followed by transferring into new well plates containing an equal volume of 0.1 N NaOH. TRAP activity was measured at λ = 405 nm and expressed as percent of that of untreated control. Data represented the mean±SEM(*P<0.05). Number of samples used is showed within the parentheses.

It was found that stimulation with RANKL for 5 days enhanced the number of TRAP-positive multinucleated cells and TRAP activity in differentiated RAW 264.7 cells. When cells were incubated with 50 nM NUCB2^1–83^, the osteoclastogenesis was reduced (Fig. 6, 7).

### NUCB2^1–83^ mRNA Expressed in Osteoblast and Osteoclast

**Figure 8:**
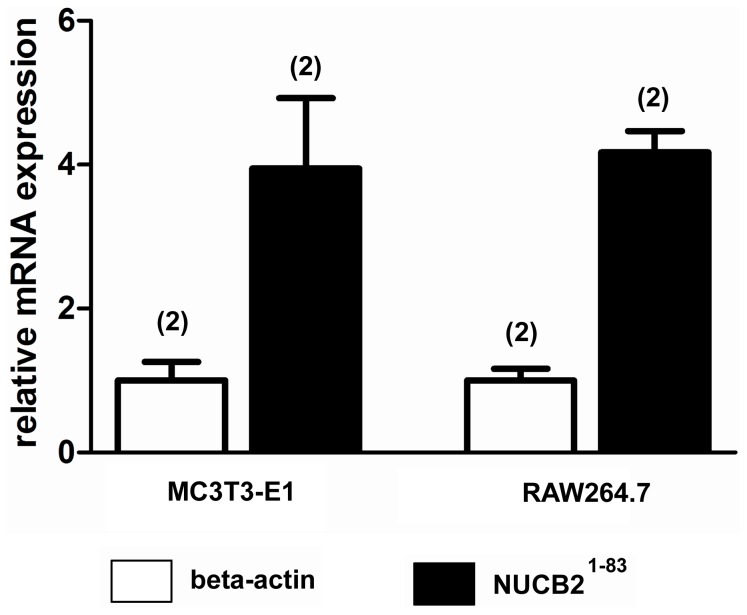
Expression of NUCB2^1–83^ mRNA in MC3T3-E1 cells and differentiated RAW 264.7 cells. The precise quantitative data were obtained by using Real-Time Quantitative PCR. The results were normalized to beta-actin, which served as a control to verify the amount of samples. Data represented the mean±SEM. Number of samples used is showed within the parentheses.

It was found that significant expression of NUCB2^1–83^ mRNA was shown in MC3T3-E1 cells and differentiated RAW264.7 cells (Fig. 8). The negative control demonstrated the base-line result.

## Discussion

The nucleobindins, NUCB1 and NUCB2, are homologous calcium and DNA binding proteins. It was reported that secreted extracellular NUCB1 might contribute in modulating the matrix maturation in bone with unknown mechanisms [12]. NUCB2^1–83^ was originally identified as an anorexigenic factor in hypothalamus which was recently reported to be anti-hyperglycemic [7]. However, it has not been reported to affect bone metabolism. In our experiments, the intravenous administration of NUCB2^1–83^ was found for the first time to increase BMD of femora and lumbar vertebrae in OVX rats.

Mouse MC3T3-E1 preosteoblastic cell line was derived from calvaria of newborn mice [13]. It has been widely applied in the investigation of mechanism underlying osteoblast differentiation as it epitomizes osteogenic differentiation and maturation *in vitro*. ALP is a representative marker for osteoblastic differentiation [14]. We discovered that NUCB2^1–83^ dramatically increased the ALP activity in MC3T3-E1 in the presence of rhBMP-2. Moreover, it increased the mineralization of MC3T3-E1. These results confirmed the promotion effect of NUCB2^1–83^ on differentiation and mineralization of osteoblasts. Meanwhile, the two mutants of NUCB2^1–83^ showed the negative results, indicating the structural importance of Arg^60^/Arg^63^ or Ser^72^. These inactive mutants also excluded any possibility of the effect caused by contaminants or experimental faults, and demonstrated that the promoting effect observed was specifically due to NUCB2^1–83^ itself.

Worthy to be noted, the promotion effect of NUCB2^1–83^ on osteoblastic differentiation was found to be dependent on rhBMP-2, since NUCB2^1–83^ itself had no direct effect on the ALP activity in the absence of rhBMP-2 (data not shown). In contrast, NUCB2^1–83^ could improve mineralization with no addition of rhBMP-2. Additionally, the expression of NUCB2^1–83^ was induced by troglitazone, an activator of peroxisome proliferator-activated receptor-γ (PPAR-γ). The activation of PPAR-γ was recognized to cause loss of bone. Therefore, the pro-osteogenic activity of NUCB2^1–83^ could also be related to PPAR mediated signaling. We presume that NUCB2^1–83^ plays differently in the different phases of osteoblast biology, which needs confirmation by further research.

Osteoclasts are bone-resorbing cells that differentiate from macrophage precursors in response to RANKL. TRAP is a important cytochemical marker of osteoclast function [15]. In our experiments, we noticed that NUCB2^1–83^ reduced formation of TRAP-positive multinucleated cells and decreased the TRAP activity in RANKL-treated RAW 264.7 cells. These results confirmed the inhibitory effect of NUCB2^1–83^ on osteoclastogenesis of murine RAW 264.7 macrophages.

Taken together, NUCB2^1–83^ promotes differentiation and mineralization of osteoblasts *in vivo and in vitro*, as well as inhibits osteoclast differentiation, indicating its important roles in metabolic control of bone. It is warranted that further studies on NUCB2^1–83^ would be valuable for the treatment of bone metabolic diseases especially for osteoporosis.
